# Effect of Water Supplementation on Cognitive Performances and Mood among Male College Students in Cangzhou, China: Study Protocol of a Randomized Controlled Trial

**DOI:** 10.3390/ijerph14090966

**Published:** 2017-08-27

**Authors:** Na Zhang, Songming Du, Zhenchuang Tang, Mengqi Zheng, Guansheng Ma

**Affiliations:** 1 Department of Nutrition and Food Hygiene, School of Public Health, Peking University, 38 Xue Yuan Road, Hai Dian District, Beijing 100191, China; ziqingxuanping@126.com; 2 Laboratory of Toxicological Research and Risk Assessment for Food Safety, Peking University, 38 Xue Yuan Road, Hai Dian District, Beijing 100191, China; 3 Chinese Nutrition Society, 6 Guang An Men Nei Street, Xi Cheng District, Beijing 100053, China; dusm9709@126.com; 4 Institute of Food and Nutrition Development, Ministry of Agriculture, 12 Zhong Guan Cun Nan Street, Hai Dian District, Beijing 100081, China; tangzhenchuang@126.com; 5 National Institute for Nutrition and Health, Chinese Center for Disease Control and Prevention, 29 Nan Wei Road, Xi Chen District, Beijing 100050, China; zhengmq93@163.com

**Keywords:** water supplementation, hydration, cognitive performances

## Abstract

*Introduction*: Water accounts for about 75% of brain mass. Cognitive performances and mood may be impaired by hypohydration and improved by water supplementation. Two surveys conducted in China demonstrated that a large proportion of adults and children drank less fluid than the amounts recommended by the Chinese Nutrition Society. The association between hypohydration and cognitive performance has not been reported in China. The purpose of this study is to explore the effect of water supplementation on cognitive performances and mood among male college students in Cangzhou, China. *Methods and Analysis*: A randomized controlled trial is designed to test the hypothesis. A total of 68 male college students aged 18–25 years will be recruited and randomly assigned into water-supplementation group (WS group, *n* = 34) and no water-supplementation group (NW group, *n* = 34) after an overnight fasting, i.e., without eating foods and drinking fluid for 12 h. The first morning urine will be collected to determine urine osmolality on the water supplementation day. Cognitive performances and mood will be performed before water supplementation by researchers with questionnaire. Subjects in the WS group will drink 400 mL purified water within 5 min, while those in NW group will not drink any fluid. One hour later, urine will be collected and urine osmolality, cognitive performances and mood will be measured again. Mixed model of repeated measures ANOVA will be used to investigate the effect of water supplementation on cognitive performances. The study would provide information about the benefit of water supplementation on cognitive performances. *Ethics and Dissemination*: The study protocol is reviewed and approved by the Ethical Review Committee of the Chinese Nutrition Society. Ethical approval project identification code is CNS-2015-001. Results will be published according to the CONSORT statement and will be reported in peer-reviewed journals. *Trial registration*: Chinese clinical trial registry. Identifier: ChiCTR-IOR-15007020. Registry name “The effect of hydration on cognitive performance”.

## 1. Introduction

Water is the main component of human body and is crucial for human survival. Water has functions of participating in metabolism, modulating normal osmotic pressure and maintaining electrolyte balance [1]. In addition, heat generated in the process of metabolism can be absorbed by water so as to avoid rapid increase in body temperature and there is also an important way to maintain a constant body temperature through evaporating sweat from skin surface [2]. Furthermore, water accounts for about 75% of brain mass. It is indicated that hypohydration induced by deficiency of total body water [3] may impair cognitive performances and mood, water supplementation may improve cognitive performances and mood. 

A few studies found the associations between hypohydration and cognitive performances. Cian et al. indicated that heat stress and exercise-induced hypohydration impaired perceptive discrimination, psycho-motor skills, and short-term memory and had adverse effect on fatigue [4,5]. Lieberman et al. revealed that military exercise in heat had adverse effect on cognitive performances and mood [6]. Baker et al. demonstrated that vigilance-related attention was impaired by hypohydration after a basketball game in the heat [7]. D’anci et al. showed that digit span performance, vigilance attention, choice reaction decrements resulting from mild hypohydration induced by exercise and fluid restriction [8]. Ganio et al. showed that the mild hypohydration induced by combination of exercise and diuretics impaired vigilance and working memory, and increased tension/anxiety and fatigue [9]. Armstrong et al. also reported that hypohydration in females can increased perception of task difficulty and degraded mood [10]. However, some studies obtained inconsistent results. Sharma et al., Bandelow et al. and Ely et al. showed that no statistically significant change in cognitive performances was observed under hypohydration induce by heat stress and exercise [11,12,13]. In most studies, a state of hypohydration was induced through heat stress, physical activity, or diuretics, and so on. However, heat stress and physical activity themselves may have impacts on cognitive performances. Hypohydration state in free-living life was studied less, which need further studies.

Another important issue is whether water supplementation have beneficial effects of on cognitive performances. Rogers et al. showed that cognitive performances improved depending on thirst after water supplementation [14]. Edmonds et al. also revealed that subjective thirst moderated the positive effect of water supplementation on speed of responding [15]. In another study of Edmonds et al., it was reported that water supplementation had effect on cognitive performances, no matter with either expectancies or mood [16,17,18]. Fadda et al. suggested a beneficial effect of water supplementation on short-term memory. Benefer et al. reported that water intake were associated with post-exercise cognitive performance (short-term memory) [19]. However, paradoxically, Neave et al. and Benton et al. demonstrated that water supplementation had no effect on cognitive performances. Backes et al. demonstrated that there was a lack of facilitation of cognition in the 500 mL water supplementation condition other than in the 150 mL condition [20]. Different amount of water supplementation, different types of cognitive performances and different duration after water supplementation may cause conflict results, which need more studies with appropriate study design.

In China, there are no studies about the effects of hypohydration state and water supplementation on cognitive performances. Only two surveys about fluid intake were conducted. In 2009, Ma et al. conducted a fluid intake survey among 1483 adults from four cities in China, it was found that approximately 32% of the subjects drank less water than the 1200 mL/day recommended by the Chinese Nutrition Society [21]. In another survey among 5868 primary and middle school students from the same four cities [22], it was reported that nearly two-thirds of the subjects drank less than the recommended amount. The results of two above-mentioned surveys imply that large proportion of Chinese residents may be in a hypohydration state, and their cognitive may be impaired. One hypothesis is that hypohydration state will impair cognitive performances and mood. Another hypothesis is that water supplementation will improve hydration state, cognitive performances and mood. The objectives of this study are, firstly, to evaluate the effect of water supplementation on cognitive performances, secondly, to explore its effect on mood among college students, and finally, to raise the awareness of keeping adequate hydration state and promote water-related education in China.

## 2. Methods and Analysis

### 2.1. Study Hypothesis

One hypothesis is that hypohydration state will impair cognitive performances and mood. Another hypothesis is that water supplementation will improve hydration state, cognitive performances and mood.

### 2.2. Study Design and Assignment of Interventions

**Figure 1:**
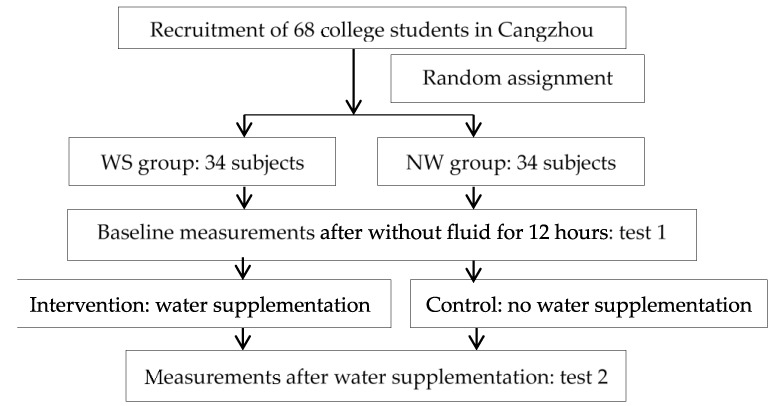
Flow diagram of study design.

This is a randomized controlled trial. After being encoded according to student ID, recruited subjects will be randomly assigned to the water-supplementation group (WS group, 34) and no water-supplementation group (NW group, 34) using random number table to generate random sequence by investigators. Measurements on blood pressure, blood glucose, urine osmolality, urine sodium concentration, urine specific gravity, test of subjective sensation of satiety, hunger, sleepiness and thirst, mood and cognitive performances will be performed before and after water supplementation (Figure 1). All items from the World Health Organization Trial Registration Data Set are shown in Table 1.

### 2.3. Sample Size Calculation

The variable used for the calculation of sample size is digit span. Digit span is used to assess short-term memory. It was reported that the average values of digit span for subjects with water supplementation and without water supplementation were 10.5 and 11.5, respectively [18]. The values was taken as reference for our study and 80% power is set. SAS 9.2 (SAS Institute Inc., Cary, NC, USA) is used to calculate the sample size. Only male students will be studied, for the reason that women’s menstrual cycle may have impacts on urine indicators and cognitive performances. In addition, 10% drop-out rate is taken into account. Eventually, a total of 68 subjects will be needed. 

### 2.4. Subjects

Subjects will be recruited from freshman and sophomore years in one college in Cangzhou, Hebei province of China, by the method of online recruitment advertisements. Inclusion criteria: aged between 18 and 25 years; male; being in healthy state. Exclusion criteria: aged <18 years or >25 years; smoking, habitual alcohol (>20 g/day) consumption [23] or intensive physical exercise, or with the diseases of cognitive disorder, diabetes, gastrointestinal tract disease, oral disease, kidney disease, other chronic diseases and metabolic diseases. 

### 2.5. Ethics

The study protocol has been approved by the Ethical Review Committee of the Chinese Nutrition Society. Ethical approval project identification code is CNS-2015-001. The study will be conducted according to the guidelines of the Declaration of Helsinki. Prior to the study, all subjects will read and sign the informed consent form.

### 2.6. Study Procedure

A total of nine days will be needed to complete the study.

#### 2.6.1. Assessment of Total Drinking Fluid

In order to assess the daily total drinking fluid, all subjects will complete a self-administrative 7-day 24-h fluid intake record questionnaire after training, during the period of day 1 to day 7. The type and the amounts of drinking fluid for each time will be recorded and measured by a cup to the nearest of 10 mL. Subjects will pour fluid into the cups to measure the amount before they drink the bottle water, sugar sweetened beverages, milk, etc. The method of 7-day 24-h fluid intake record has been used in previous studies and has been proved to be reliable [16,17,18]. 

#### 2.6.2. Assessment of Fluid from Foods

All foods eaten by subjects will be weighed for three consecutive days during the seven days (including two weekdays and one weekend day) to calculate the daily fluid intake from foods. 

#### 2.6.3. Temperature and Humidity

The temperature and humidity of indoor and outdoor at the study site will be recorded at 10:00 a.m., 2:00 p.m. and 8:00 p.m. in the nine days with a temperature hygrometer.

#### 2.6.4. Assessment of Baseline Indicators

On day 8 (the day before the supplementation day), all subjects will be asked to be fast without any foods and fluid from 8:00 p.m. They will be asked to sleep before 11 PM in the evening and not urinate after sleep until getting up in the next morning to collect first urine samples. In addition, they will be asked not to do high intensity exercise before the test. Anyone who fails to meet the above requirements needs to let investigators know. The first morning urine samples in the water supplementation day will be collected in sterile disposable urine sample cup by themselves and will be tested immediately by professional lab technicians in hospital (test 1).

#### 2.6.5. Groups of Subjects

On day 9 (the water supplementation day), all subjects will be asked to arrive at the designated place at 8:00 AM. After being encoded according to student ID, subjects will be randomly assigned to the water-supplementation group (WS group, 34) and no water-supplementation group (NW group, 34) using random number table to generate random sequence. Baseline height, weight, waist circumferences, blood pressure and blood glucose will be measured (test 1: before water supplementation) following the standardized procedures. The first morning urine samples will also be collected and tested. At 8:30 AM, cognitive performances, mood and subjective sensation of satiety, hunger, sleepiness and thirst will be tested (test 1).

#### 2.6.6. Water Supplementation

**Figure 2:**
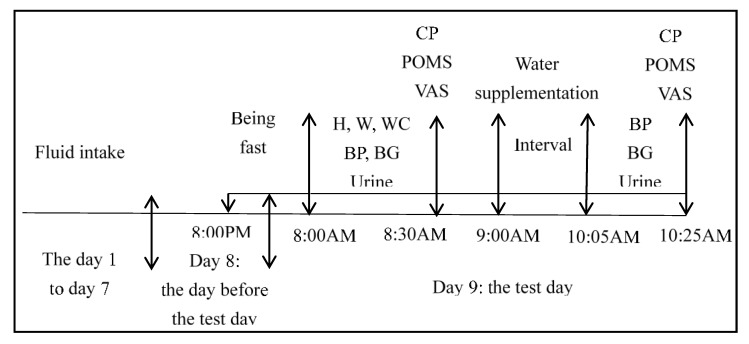
The study procedure. Note: H (Height); W (Weight); WC (Waist circumference); BP (Blood pressure); BG (Blood glucose); CP (Cognitive performances); POMS (Profile of Mood States); VAS (Visual Analogue Scales).

At 9:00 AM, subjects in the WS group will drink 400 mL purified water within 5 min with uniform cups in one designated place, while those in NW group will not drink any water or beverages in another designated place. Subjects in NW group will not be told the intervention of WS group. During the interval before the next test, all subjects will be asked to sit at their own seat without physical activity. They will be asked not to eat foods, drink water/beverages, and urinate. One hour after water supplementation (at 10:05 AM), blood pressure and blood glucose will be measured again in designated place (test 2: after water supplementation). Urine samples will be collected, stored and tested again (test 2). At 10:25 AM, cognitive performances, mood and subjective sensation of satiety, hunger, sleepiness and thirst will be measured again (test 2). Temperature and humidity will be recorded at 8:00 AM. During the period of the study, subjects can quit at any time for side effects, such as syncope. The study procedure is shown in Figure 2.

### 2.7. Blinding of Data Analysis

Data analysts will analyze the data without being informed which group is water supplementation group or no water supplementation group. The names of groups will be coded as A and B. After data analysis and the finish of summary report, data analysts will be informed the group code.

The schedule of enrolment, interventions, and assessments is shown in Table 2.

### 2.8. Assessment of Fluid Intake

Daily total drinking fluid of the subjects will be collected using a 7-day 24-h fluid intake record by themselves after standardized training to use the fluid intake record correctly. The amount of drinking fluid for each time will be measured by a cup with the nearest of 10 mL. Subjects will pour fluid into the cups to measure the amount when they drink liquid. The method of 7-day 24-h fluid intake record has been used in previous studies and was proved to be reliable. All sources of drinking fluid will be included in, such as boiled water, bottle water, brewed tea, tea drinks, carbonated soft drinks, fruit and vegetable drinks, protein drinks, functional drinks, solid drinks and so on. The type, place, time, and amount of fluid will also be recorded by subjects. To ensure the completeness of 7-day 24-h fluid intake record, questionnaire will be reviewed by investigators every day.

Fluid intake from foods will be assessed with duplicate portion method. All foods consumed by the subjects will be weighted for three consecutive days (including two weekdays and one weekend day). Samples of all foods will be collected and measured according to the national standard of GB 5009.3-2010 by professionals in Analysis Laboratory of Beijing Nutrition Resources Institute. (Daily total fluid intake (mL) = Daily total drinking fluid (mL) + Daily fluid intake from foods (mL)).

### 2.9. Anthropometric Measurements

Height (H) will be measured twice to the nearest 0.1 cm and weight (W) will be measured twice to the nearest 0.1 kg while wearing light clothing and no footwear by trained investigators following standardized procedures with height-weight meter (HDM-300; Huaju, Yiwu, Zhejiang, China) in a standardized time (during 8:00 AM to 8:30 AM in the water supplementation day). 

Waist circumference (WC) will be measured twice at the midpoint between the bottom of the rib cage and the top of the iliac crest at the end of exhalation with the subject standing without clothing covering the waist area to the nearest 0.1 cm by trained investigators with a MyoTape waistline measurer in a standardized time (during 8:00 AM to 8:30 AM in the water supplementation day). (BMI: weight (kg)/height squared (m)]; body surface area (m^2^): (weight (kg)^0.425^ × height (cm)^0.725^) × 0.007184 [24]). 

Blood pressure (BP) will be measured twice to the nearest 2 mmHg by a nurse with electronic sphygmomanometer (HEM-7051; Omrom, Dalian, Liaoning, China) at a standardized time (during 8:00 AM to 8:30 AM in the water supplementation day). Two measurements will be taken after 2-min intervals.

Blood glucose (BG) will be determined once with fingertip blood of the index finger by a professional nurse with a glucometer (One Touch UltraEasy; Johnson, Shenzhen, China) at a standardized time (between 8:00 AM to 8:30 AM on the water supplementation day).

### 2.10. Assessment of Urine Biomarkers

Urine samples will be collected twice in disposable flexible packaging plastic bags by the subjects in the day 9 (the water supplementation day). Urine samples will be stored at +4 °C before assessment. Urine volume will be measured to the nearest 0.1 kg with a desktop electronic scale (YP20001; SPC; Shanghai, China). Urine osmolality will be tested using an osmotic pressure molar concentration meter (SMC 30C; Tianhe, Tianjin, China) with the freezing-point method. Urine-specific gravity (USG) will be tested using an automatic urinary sediment analyzer (FUS-200; Dirui, Changchun, China) with the uric dry-chemistry method. Urine pH will be measured using a urine analyzer (Uritest-180, Uritest; Guilin, China) with the reflective photoelectric colorimetry method. The urine concentrations of potassium (K), sodium (Na) and chloride (Cl) will be tested using an automatic biochemical analyzer (Cobas C501; Roche, Basel, Switzerland) with the ion-selective electrode potentiometer method.

### 2.11. Definition of Hypohydration

The balance between water outputs and water inputs defines hydration state [25]. Hypohydration occurs when water inputs is insufficient to replace free water outputs, which is defined as the urine osmolality is greater than 800 mOsm/kg [26]. 

### 2.12. Visual Analogue Scales (VAS) for Subjective Sensation

VAS is a self-rated 10-cm-line designed to quantitatively measure subjective sensation of satiety, hunger [27], sleepiness [28] and thirst [29]. On the line of VAS, the labels “not at all” and “extremely” will be anchored at the beginning and end. Subjects will be instructed to place a vertical line on it corresponding to their current degree of feeling. The range of scores varies between 0 and 10 according to the length of the line. The higher score means the higher level of corresponding subjective sensation.

### 2.13. Profile of Mood States (POMS)

The self-rating mood questionnaire consisted of seven subscales, 40 adjectives that measured tension, depression, fatigue, vigor, confusion, anger, esteem-related affect and total mood disturbance [TMD: (tension + depression + anger + fatigue + confusion) − (vigor + esteem-related affect) + 100]. TMD is a global estimate of affective state [30]. Method of evaluating score: choose one scale for each adjective which conforms to own situation from five scales (0 not at all, 1 a little, 2 moderately, 3 quite a bit, 4 extremely). All answers will be analyzed through a 5-point rating.

### 2.14. Cognitive performances (CP)

*Digit span forward and backward*: This will be used to assess short-term memory. A list of random numbers will be read out loudly at the rate of one per second by trained investigators. After reading a digit sequence, subjects will be asked to recall the digits in forward or backward order. The digits sequence will begin with three numbers, which should be without recognizable digit patterns on two consecutive trials of the same length. Two incorrect repetitions result in termination of the test. Digit span forward, for example, if the investigator reads ‘‘1 2 3’’, the participant will repeat ‘‘1 2 3’’. Digit span backward, for example, if the investigator reads ‘‘1 2 3’’, the participant will repeat ‘‘3 2 1’’.The number of digit without error will be counted as score respectively for digit span forward and backward [18]. Total score will be used to assess overall performances (Total score = forward score + backward score).

*Digit symbol substitution test*: This test will be used to assess digital decoding ability, visual memory, visual attention, and operating speed. It consists of nine digit-symbol pairs followed by a list of digits (total 90) (for example, 1/_, 2/⊥ ... 7/Λ, 8/X, 9/=). Subjects will be asked to write down the corresponding symbol under each digit as fast as possible within the allowed time (90 s). The number of correct symbols will be measured for score [31].

*Dose-work test*: It will be used to assess the ability of sustained attention. An Aventura Karimov table [32] will be used to evaluate the quality and quantity of dose-work. Subjects will be asked to cross the letter B behind the letter H line by line in 2 min—in an A4 format page filled with eight different letters (A, B, C, E, H, K, N, X) in a random order; total 1200 characters are on one page; each letter appear 150 times. (Reading speed (n/min) = number of already-read letters (n)/2 (min); error rate (%) = number of errors (n)/number of already-read letters (n) × 100%; mental work ability index (IMC) = number of already-read letters (n)/2 (min) × (number of letters had to be crossed − number of errors)/number of letters had to be crossed). 

*Creativity*: Given four kinds of description, such as “usually red”, “usually round”, “usually very noisy”, “usually have wheels”, subjects will be asked to write down the objects as much as possible in accordance with the corresponding description without same type in 5 min [33]. (C-score = log2 (1/P_1_) + log2 (1/P_2_) +…+ log2 (1/P_n_), P= the probability that the answer being written down by a randomly selected child.)

*Logic*: Subjects will be asked to judge the correctness of 25 logic statements. For example: A follows B in “AB” (false), B is not preceded by A in “BA” (true). The number of already-completed correct number is measured for score [34]. (Correct rate (%) = right numbers (n)/already-completed numbers (n) × 100%).

### 2.15. The Possible Potential Harm of the Study on Subjects

Subjects will be fasting without water, which may induce hypohydration. Health effects of insufficient water intake include emergency of the sensation of thirst, decreased physical activity, increased plasma osmotic pressure and reduction of urine. If subjects in the study appear side effects and harmful responses, professional medical staff will give treatment timely. 

Subjects can withdraw from the trial at any time following their own requirements. If subjects in the study appear side effects and harmful responses, they can withdraw the trial freely and be given timely treatment by researchers.

### 2.16. Auditing of Trial Conduction

Trial conduction will be audited by competent authority in the college every day, and the process will be independent from investigators and the sponsor.

### 2.17. Strategies to Improve Adherence to Intervention Protocols

The principles and requirements of the study will be fully explained and anyone who fails needs to let researchers known. In order to improve adherence, the researchers will meet and communicate with subjects every day in order to find problems and handle them timely. The reasons and time for drop-out will also be recorded in detail. The urine biomarkers will be evaluated by Data monitoring committee (DMC) once a day for monitoring adherence. 

### 2.18. Confidentiality

Researchers will keep the information confidential for the subjects. The names of subjects will be coded as study ID during, and after the trial.

### 2.19. Data Collection, Entry and Statistical Analysis

Data collection: Researchers will be trained about the trial process, the use of questionnaires and laboratory tests. Subjects will be trained to be familiar with the related questionnaire and the requirements in the study. Some indicators are measured twice to ensure their accuracy, such as height, weight, waist circumstance and blood pressure. If subject withdraw the trial, his data will not be analyzed. All the questionnaires will be stored for regularly checked and rechecked by data monitoring committee. 

*Data entry*: All data will be recorded and entered into Epi Data 3.1 twice to ensure the accuracy by trained researchers. 

*Statistical analysis*: SAS 9.2 (SAS Institute Inc., Cary, NC, USA) will be used for statistical analyses. Quantitative parameters for subjects will be presented as mean ± standard deviation; binary classification data (hypohydration) will be presented as n ± percentage. Quantitative parameters at test 1 between two groups will be compared with the method of Student’s *t*-test (for normally distribution) and Mann-Whitney *u*-test (for abnormally distribution); binary classification data (hypohydration) at test 1 between two groups will be compared with the method of Chi-square test. Mixed model of repeated measures (ANOVA) will be used to investigate the effect of water supplementation on changes in urine index, mood and cognitive performances, whilst covarying age, BMI, hunger, satiety, sleepiness, blood pressure, blood glucose, body area and fluid intake. Significance levels will be set at 0.05 (*p* < 0.05, 2-tailed) with 95% confidence intervals (95% CI). Data of subjects without compliance will be included in data analysis.

### 2.20. Monitoring of Data Management

DMC is composed by six members (two clinicians, one statistician, one ethicist, two nutritionists) with no competing interests, who are independent of the entity conducting the trial and independent from the sponsor. DMC will have a meeting once a day to review the procedure and data of the study. The main contents of the meeting are as follows: evaluation of results (safety concerns, outstanding benefit, and futility) to decide the continuation or termination of the study; examination of the quality and completeness of the data; supervision of the quality control of the study procedure; record of follow-up process and supervision of data entry and analysis. The Methods section of this manuscript followed the SPIRIT guidelines.

## 3. Discussion

Adequate hydration is crucial for human survival and homeostasis, including maintaining brain function. Hypohydration caused by a deficiency of total body water, may impair cognitive performances and mood. However, the related studies did not get consistent conclusion. There are some reasons for the inconsistency. For example, hypohydration was induced by heat stress, fluid restriction, exercise, diuretics, or combinations of above methods in these studies. However, the above methods of inducing hypohydration themselves may affect cognitive performances. Tomporowski et al. reported that short-term memory performance improved after exercise, due to the metabolic arousal caused by strenuous physical activity [35]; Trezza et al. pointed that heat stress may impair cognitive performances due to the unpleasant sensation [36]. Different cognitive testing methods, different amount of water supplementation and different degree of hypohydration, and other confounding factors may also cause inconsistent results. It is important to make the related results of different studies comparable and further study is needed to verify the interaction with a good study design. In our study, a randomized controlled design will be used to evaluate the effect of water supplementation on cognitive performances and mood. 

The biggest challenge we will face with is the adherence of subjects and quality control. In the study, subjects will be asked to be fast without food and water for 12 h. What is more difficult, subjects will not be allowed to urinate after sleep until the next morning. Therefore, the principles and requirements of the study will be fully explained and anyone who fails needs to let researchers known. In order to enhance adherence, the researchers will meet and communicate with subjects every day in order to find problems and handle them timely. The reasons and time for drop-out will also be recorded in detail. To better control the quality of the study, each measurement will be monitored and managed by trained researchers. Two large surveys of drinking water among adults and school children from four cities in China has been conducted by our team, which provides useful experience and technical support for the assessment of fluid intake in our study. Anthropometric indexes, such as heights, weights and waist circumferences, will be measured twice by trained researchers to ensure the stability and reliability of data. Physiological indexes, such as blood pressure, blood glucose, urine osmolality, urine sodium concentration, urine specific gravity, will be determined by professional nurse and lab technicians with abundant clinical experience in hospital. In addition, urine will be immediately tested after collection, in order to reduce the effect of long time storage on results. Questionnaire of cognitive performances has been used in previous studies by our team and the process in this study will be conducted under professional researchers under standard procedure.

The present study design has both strengths and weaknesses. In terms of strength, first, the possible confounding factors are considered relatively comprehensive. Age, BMI, hunger, satiety, sleepiness, blood pressure, blood glucose, body area and fluid intake will be measured and included as covariates to make a more accurate conclusion. Second, a randomized controlled design will be used to reduce bias. Third, hypohydration state will be induced by the simple method of fluid restriction, which are meaningful to explain its effect on cognitive performance more clearly. Fourth, subjects will be fasted for 12 h in order to eliminate the influence of dietary on the test. In most previous studies, the group without water supplementation was subjectively considered as hypohydration group, which may be not consistent with the actual hydration state. In our study, urine osmolality will be measured in order to objectively evaluate the changes of hydration state. In terms of weakness, gender differences will not be explored in our study. In addition, the effect of long-term water supplementation on cognitive performance will not be studied. The mechanism will also not be explored.

## 4. Conclusions

In summary, it is the first time the effect of water supplementation on cognitive performances and mood among college students in China is evaluated. The study should provide more useful information on the beneficial of water, which is helpful for the education related to drinking water. It can raise residents’ attention to drinking water and maintain a good hydration state among the Chinese population. 

## Figures and Tables

**Table 1:** All items from the World Health Organization Trial Registration Data Set (SPIRIT checklist, item 2b).

Data Category	Information
Registration number	ChiCTR-IOR-15007020, Chinese clinical trial registry
Registration State	1008001 Prospective registration
Public title	The effect of hydration on cognitive performance
Scientific title	The effect of hydration on cognitive performance
Approval of ethic committee	Chinese Nutrition Society biomedical ethics committee
Ethical approval project identification code	CNS2015001
Date of approved by ethic committee	1 December 2015
Study type	Interventional study
Study design	Randomized parallel controlled trial
Key inclusion and exclusion criteria	Inclusion criteria: Aged between 18~25; Male; In health state, without metabolic disease, oral diseases, and so on. Exclusion criteria: Aged < 18, or age > 25; Female; With metabolic disease, oral diseases, and other diseases.
Interventions	Water supplementation
Outcomes	Urine osmotic pressure, Blood pressure, Mood, Thirsty, Cognitive performance
Collecting samples	Urine
Recruitment state	Finished
Randomization Procedure (please state who generates the random number sequence and by what method)	Primary sponsor generates random sequence with random number table.

**Table 2:** The schedule of enrolment, interventions, and assessments.

	Enrolment	Allocation	Post-Allocation	Close-out
Timepoint	1 February 2016	14 March 2016	14 March 2016	30 March 2016
Enrolment:				
Eligibility screen	X			
Informed consent	X			
Allocation		X		
Interventions:				
Water supplementation			X	
Assessments:				
Baseline anthropometric measurements variables: blood pressure, height, weight, waist circumference	X		X	
Outcome variables: cognitive performances, mood, subjective sensation			X	X

## Abbreviations

WS group	Water-supplementation group
NW group	No water-supplementation group
H	Height
W	Weight
WC	Waist circumference
BP	Blood pressure
BG	Blood glucose
UO	Urine osmolality
TMD	Total mood disturbance
POMS	Profile of mood states
CP	Cognitive performances
VAS	Visual analogue scales
